# Decisions on statin therapy by patients’ opinions about survival gains: cross sectional survey of general practitioners

**DOI:** 10.1186/s12875-015-0288-8

**Published:** 2015-07-03

**Authors:** Peder A. Halvorsen, Olaf Gjerløw Aasland, Ivar Sønbø Kristiansen

**Affiliations:** Department of Community Medicine, UiT - The Arctic University of Norway, P.o. box 6050 Langnes, N-9037 Tromsø, Norway; LEFO - Institute for Studies of the Medical Profession, The Norwegian Medical Association, P. box 1152 Sentrum, N-0107 Oslo, Norway; Department of Health Management and Health Economics, Institute of Health and Society, University of Oslo, P. box 1089 Blindern, N-0318 Oslo, Norway

## Abstract

**Background:**

Guidelines for primary prevention of cardiovascular disease provide little guidance on how patients’ preferences should be taken into account. We wanted to explore whether general practitioners (GPs) are sensitive to patient preferences regarding survival gains from statin therapy.

**Methods:**

In a cross sectional, online survey 3,270 Norwegian GPs were presented with a 55 year old patient with an unfavourable cardiovascular risk profile. He expressed preferences for statin therapy by indicating a minimum survival gain that would be considered a substantial benefit. This survival gain varied across six versions of the vignette: 8, 4 and 2 years, and 12, 6 and 3 months, respectively. Participants were randomly allocated to one version only. We asked whether the GPs would recommend the patient to take a statin. Subsequently we asked the GPs to estimate the average survival gain of life long simvastatin therapy for patients with a similar risk profile.

**Results:**

We received 1,296 responses (40 %). Across levels of survival gains (8 years to 3 months) the proportion of GPs recommending statin therapy did not vary significantly (OR per level 1.07, 95 % CI 0.99 to 1.16). The GP’s own estimate of survival gain was a statistically significant predictor of recommending therapy (OR per year adjusted for the GPs’ age, sex, speciality attainment and number of patients listed 3.07, CI 2.55 to 3.69).

**Conclusion:**

GPs were insensitive to patient preferences regarding survival gain when recommending statin therapy. The GPs' recommendations were strongly associated with their own estimates of survival gain.

## Background

Shared decision making is recommended when treatments have benefits and harms that people value differently [1]. Arguably the decision to initiate statin therapy for primary prevention of cardiovascular disease is a preference sensitive decision [2]. Benefits in terms of gains in quality and length of life in the long run must be weighed against potential harms such as side effects and disease labelling in the present. To facilitate shared decision making, conversations between doctors and patients should clarify the benefits and risks involved as well as patients’ values and preferences [3].

Informing patients about benefits of risk reducing drug therapies is not a trivial task. Studies of lay peoples’ hypothetical decisions suggest that they may find it difficult to evaluate traditional effect measures such as relative risk reduction [4] and number needed to treat [5, 6]. Alternatively, benefits can be explained in terms of gains in life expectancy or postponement of adverse events [7], and such effect measures may be easier to evaluate [8, 9]. More recently it has been shown that patients were less likely to redeem statin prescriptions when informed about the benefits in terms of survival gains rather than absolute risk reduction [10]. Others have shown that many physicians overestimate survival gains from statin therapy, and those physicians were more likely to recommend such therapy [11, 12].

Guidelines for primary prevention of cardiovascular disease provide tools for risk calculation and decision thresholds for preventive drug therapy [13], but there is not much guidance about how patients’ values and preferences should be taken into account. We wanted to explore whether general practitioners (GPs) are sensitive to the patients’ preferences when considering statin therapy. Specifically, we tested the hypothesis that the proportion of GPs recommending such therapy would vary by the survival gain a hypothetical patient would require for the therapy to be considered worthwhile.

## Methods

### Participants

In December 2009 3,270 GPs registered with the Norwegian Medical Association were sent an e-mail asking them to participate in an online survey. We aimed to include all GPs in Norway at the time (n = 4,049, Table 1), but a random sample of GPs, who were invited to another survey taking place at the same time, were excluded. Thus a random sample comprising 81 % of all Norwegian GPs were invited. The online questionnaire was administered by the Institute for Studies of the Medical Profession, a small independent research unit hosted by the Norwegian Medical Association. Return of the questionnaire was considered as consent to participate in the study.

**Table 1:** Respondent characteristics among general practitioners randomised to six versions (A-F)^a^ of a vignette portraying a patient with an unfavourable cardiovascular risk profile

Group	Mean age	Proportion female	Mean # of patients listed	Specialty attainment
A	47	84/236 (36 %)	1222	149/237 (63 %)
B	48	77/211 (37 %)	1188	150/214 (70 %)
C	47	64/203 (32 %)	1242	138/203 (68 %)
D	48	85/225 (38 %)	1216	156/226 (69 %)
E	47	81/220 (37 %)	1175	139/220 (63 %)
F	47	78/208 (38 %)	1213	130/208 (63 %)
All Norwegian GPs n = 4,049^b^	49^c^	35%^c^	1182^c^	55%^d^

^a^The vignettes differed with respect to the survival gain the patient would require to consider simvastatin therapy worthwhile (A: 3 months, B: 6 months, C: 12 months, D: 2 years, E: 4 years, F: 8 years)

^b^Statistics Norway (http://www.ssb.no accessed 24th of March 2011)

^c^ https://helsedirektoratet.no/Documents/Statistikk%20og%20analyse/Fastlegestatistikk/Fastlegestatistikk%20hovedtallsrapport-2010.pdf

^d^ http://www.legeforeningen.no/id/18 14.04.2011

### Procedure

**Fig. 1:**
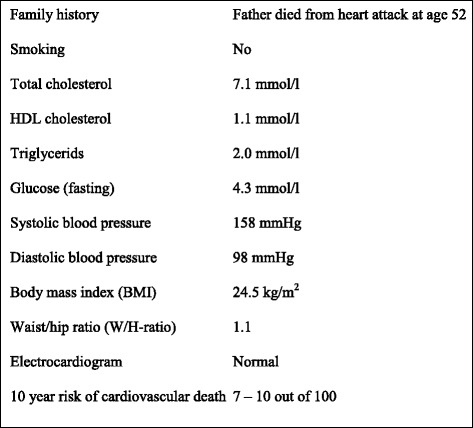
Risk profile of Mr. Hansen – a hypothetical 55 year old patient with a family history of premature coronary heart disease

The questionnaire presented Mr. Hansen, a physically fit, non-smoking male aged 55 who consulted his GP for measurement of blood pressure and cholesterol because his father had suffered a heart attack at age 52. Mr. Hansen had no symptoms, but elevated blood pressure (160/90 mmHg) and total cholesterol (7.5 mmol/liter). He received dietary advice, but after three months his risk profile was virtually unchanged (Fig. 1) with a ten year absolute risk of cardiovascular death estimated at 7 – 10 in 100 patients. According to current Norwegian guidelines statin therapy is indicated in this situation, but there was no reference to the guidelines in the scenario. At the end of the scenario Mr. Hansen stated that he was happy with his life and wanted it to last as long as possible. He would prefer achieving this through exercise and reasonably healthy diet, but would consider drug therapy if the benefit was substantial. Finally we quantified the survival gain that the patient regarded as substantial. We had six versions of the scenario, where this survival gain was set at 3, 6 and 12 months and 2, 4 and 8 years, respectively. The GPs were randomly allocated to one scenario only. We asked the GPs whether they would recommend statin therapy for Mr. Hansen. Possible response options were “certainly”, “probably”, “probably not” and “certainly not”. Subsequently they were asked to estimate the average survival gain of life long simvastatin therapy for patients like Mr. Hansen. Possible response categories were <12, 12, 18, 24, 30, 36, 42, 48 and >48 months, respectively. Due to the low number of respondents in some of the categories we collapsed these into five groups in the analysis (<12 months, 12 or 18, 24 or 30, 36 or 42 and 48 or more months, respectively). For other study purposes the questionnaire had questions about what professional activities the GPs considered meaningful and preferences for practice organisation and remuneration (presented elsewhere, [14, 15]).

### Outcome measures

Our primary hypothesis was that more GPs would recommend statin therapy when the survival gain that Mr. Hansen required was shorter. Consequently, the proportion of GPs recommending statin therapy was our primary outcome measure. Although the survey provided the GPs with a 4 graded response, we dichotomized this variable when we analyzed the survey data so that “certainly” and “probably” were counted as “yes”, whereas “probably not” and “certainly not” were counted as ”no”. Analysis of this variable as a 4-point response instead of as a dichotomous response yielded similar results, but for ease of understanding, we present the results with the responses grouped as described. We also tested whether recommending statin therapy was associated with the GPs’ estimate of survival gain for patients like Mr. Hansen. For this analysis we included age, sex, speciality attainment and number of patients listed as potential confounders.

### Statistical analysis

We used logistic regression analyses to test for linear trend across response options. Power calculation was not performed as we aimed for a large sample for other study purposes. SPSS (19.0) was used for data analysis. In Norway studies like ours do not require evaluation by an ethical research committee, but the study was approved by the Norwegian Social Science Data Services, which is the privacy ombudsman for all Norwegian universities as well as the Institute for Studies of the Medical Profession of the Norwegian Medical Association.

## Results

### Study sample

Of the 3,270 GPs invited 1,308 returned the online questionnaire, and 1,296 provided a recommendation regarding statin therapy for Mr Hansen (response rate 40 %). The respondents were fairly representative of Norwegian GPs with respect to age (mean 47 years, 95 % CI 46 to 48), sex (36 % females, CI 33 to 39) and number of patients listed (mean 1209, CI 1189 to 1229), although the total population of Norwegian GPs was slightly older (mean 49 years) and has slightly fewer patients listed (mean 1182, see Table 1). The proportion with specialty attainment was somewhat higher among the respondents (66 %, CI 63 to 68) than among Norwegian GPs in general (55 %).

### Responses to the scenarios

Across the six levels of survival gains that Mr. Hansen required to regard the benefit as worthwhile (8 years to 3 months), the proportions of GPs recommending statin therapy varied from 76 % to 87 %. A test for linear trend across the levels was not statistically significant (OR per level 1.07, CI 0.99 to 1.16, Table 2). The average survival gain of simvastatin therapy for patients like Mr Smith was correctly estimated at <12 months by 25 % of the GPs. About half of these GPs recommended statin therapy for Mr Hansen, compared to more than 80 % of GPs who thought the average survival gain was 12 months or more (Table 2). In logistic regression analysis the GP’s estimate of survival gain was a statistically significant predictor of recommending statin therapy for Mr Hansen. The OR adjusted for the GPs’ age, sex, speciality attainment, and number of patients listed, was 3.07 (CI 2.55 to 3.69) per year (Table 2). There were no statistically significant associations between recommending statin therapy and age, sex, specialty attainment and number of patients listed, respectively (data not shown).

**Table 2:** Proportion of GPs recommending simvastatin therapy by patients’ required survival gain to make therapy worthwhile, and physicians’ perception of survival gain

Variable	Proportion (%)	Odds ratio for trend (95 % CI)
Patient’s required survival gain		1.07 (0.99 to 1.16)^a^
8 years	170/205 (83)	
4 years	167/219 (76)	
2 years	173/222 (78)	
12 months	165/203 (81)	
6 months	168/213 (79)	
3 months	204/234 (87)	
Physician’s perception of survival gain		3.07 (2.55 to 3.69)^b^
<12 months	166/328 (51)	
12 or 18 months	260/317 (82)	
24 or 30 months	211/228 (93)	
36 or 42 months	117/122 (96)	
48 months or more	269/275 (98)	

^a^n = 1,296

^b^Adjusted for the GPs’ age, sex, specialty attainment and number of patients listed. n = 1,217 due to missing responses

## Discussion

### Summary

We observed that when considering statin therapy GPs were insensitive to a hypothetical patient’s preferences in terms of the survival gain required to make therapy worthwhile. The GPs’ recommendations were strongly associated with their own estimates of average survival gain for such patients, and the majority of GPs overestimated the survival gain.

### Strengths and limitations

The main strengths of this study are the large and fairly representative sample of Norwegian GPs and the randomised design. Although our response rate was modest (40 %), it is comparable to other online surveys of busy clinicians [16]. However, there are several limitations. First, this was a study of hypothetical as opposed to real life recommendations. Whether vignette studies like ours may be representative of real life practice has been a long standing concern [17], although there are some studies in support of their validity [18–21]. Second, each GP was presented with only one scenario. Offering more scenarios could have reminded the GPs that patients differ, and perhaps increased their sensitivity to patient preferences. Third, the vignette did probably not capture all that matters when GPs and patients consider statin therapy for primary prevention. For example, in addition to gains in life expectancy, delay in onset of symptoms may also be important. Others have shown that personal experience with cardiovascular disease may be as important as medication effectiveness, and that possible side effects, general dislike of taking medication and preferences for life style changes may be important reasons to decline preventive drug therapies [22]. Furthermore it is probably unusual for patients to state preferences in terms survival gains, and GPs may not be familiar with this benefit measure.

### Comparison with existing literature

In a previous study Lytsy and co-workers [12] also observed that GPs tended to overestimate the survival gain of statin therapy, and that those who did were more likely to recommend therapy. Our study adds to this evidence by introducing patient preferences in the study. Surveys of lay people suggest that when risk reduction is explained in terms of survival gain it may be easier to evaluate compared to traditional measures for risk reductions [8, 9]. Also, both a survey [23] and a randomised clinical trial suggest that the amount of survival gain from statin therapy matters to patients [10]. Although such effect measures are perhaps not much used in clinical practice yet, tables of gains in life expectancy tailored to individual risk profiles have been published [7, 24].

### Implications for practice and research

Our study suggests that the GPs’ own perceptions of benefit get more weight than patient preferences when considering statin therapy for primary prevention. Although potentially in conflict with the practice of shared decision making, this is not necessarily wrong. If, however, these recommendations are based on inflated perceptions of benefit, it would raise some concern. It is noteworthy that although current Norwegian guidelines recommend statin therapy for patients like Mr. Hansen, GPs who correctly estimated the survival gain at less than 12 months [7, 24] were less likely to comply with the guidelines. Future studies could explore whether GPs are sensitive to the patients’ assessment of treatment effectiveness when expressed in terms of more traditional effect measures such as absolute or relative risk reduction, or in terms of qualitative statements such as “this treatment effect is good (or bad)”. Besides varying preferences for treatment effectiveness, patients may differ with respect to preferences regarding risk of side effects [22, 25], taking pills [22], desire to participate in decision making [25], and also with respect to the social context in which decisions are made [26]. Further insight into whether and how GPs take the patient perspective [26] into account when considering statin therapy could be important to understand enablers and barriers for shared decision making in this area.

## Conclusion

Our findings suggest that GPs may be insensitive to patient preferences regarding survival gain when recommending statin therapy. On the other hand the GPs' own estimates of survival gain were strongly associated with their recommendations.

## Abbreviations

CI: Confidence interval
GP: General practitioner
OR: Odds ratio
SPSS: Statistical package for the social sciences
